# Liver sinusoidal endothelial cells contribute to the uptake and degradation of entero bacterial viruses

**DOI:** 10.1038/s41598-020-57652-0

**Published:** 2020-01-21

**Authors:** Cristina I. Øie, Deanna L. Wolfson, Tanji Yasunori, Gianina Dumitriu, Karen K. Sørensen, Peter A. McCourt, Balpreet S. Ahluwalia, Bård Smedsrød

**Affiliations:** 1 Department of Physics and Technology, Optical Nanoscopy Research Group, Faculty of Science and Technology, UiT-The Arctic University of Norway, 9037 Tromsø, Norway; 2 Department of Biotechnology, Tokyo Institute of Technology, 226-8501 Yokohama, Japan; 3 Department of Medical Biology, Vascular Biology Research Group, Faculty of Health Sciences, UiT-The Arctic University of Norway, 9037 Tromsø, Norway; 4 Present Address: Department of Medical Biology, Vascular Biology Research Group, Faculty of Health Sciences, UiT-The Arctic University of Norway, 9037 Tromsø, Norway

**Keywords:** Biological techniques, Cell biology

## Abstract

The liver is constantly exposed to dietary antigens, viruses, and bacterial products with inflammatory potential. For decades cellular uptake of virus has been studied in connection with infection, while the few studies designed to look into clearance mechanisms focused mainly on the role of macrophages. In recent years, attention has been directed towards the liver sinusoidal endothelial cells (LSECs), which play a central role in liver innate immunity by their ability to scavenge pathogen- and damage-associated molecular patterns. Every day our bodies are exposed to billions of gut-derived pathogens which must be efficiently removed from the circulation to prevent inflammatory and/or immune reactions in other vascular beds. Here, we have used GFP-labelled Enterobacteria phage T4 (GFP-T4-phage) as a model virus to study the viral scavenging function and metabolism in LSECs. The uptake of GFP-T4-phages was followed in real-time using deconvolution microscopy, and LSEC identity confirmed by visualization of fenestrae using structured illumination microscopy. By combining these imaging modalities with quantitative uptake and inhibition studies of radiolabelled GFP-T4-phages, we demonstrate that the bacteriophages are effectively degraded in the lysosomal compartment. Due to their high ability to take up and degrade circulating bacteriophages the LSECs may act as a primary anti-viral defence mechanism.

## Introduction

Bacterial viruses, or bacteriophages are among the most numerous biological entities within the human body, capable of stimulating humoral immune responses and inducing anti-phage antibodies^1,2^. Containing an impressive 2 × 10^12^ phage particles^3^ the gastrointestinal tract is the natural reservoir of phages in humans^4,5^. From here, they can spread throughout the body via different mechanisms, e.g. intestinal epithelial damage or “leaky gut”^6^, and transcytosis^7^. It is estimated that 31 × 10^9^ phage particles are transcytosed across the epithelial cell layers of the gut into the human body every day^7^, and this number rises in patients with increased gut permeability. Aberrant transcytosis may contribute to enhanced immune responses, allergic reactions, and inflammatory diseases. In these conditions, the phages that leak into the blood stream must be efficiently removed to prevent inflammatory and/or immune reactions in other vascular beds. While cellular entrance of virus and virus-like particles in host cells have been much studied, few studies were designed to look into general clearance mechanisms for circulating virus particles, including those viruses that gain access to the general circulation from the gut in states such as inflammation/epithelial damage^8^. Previous studies have identified the liver as a central player in clearance of blood-borne pathogens^9–11^. Due to the traditional view that the clearance of material from the circulation is mainly a function of the liver macrophages (or Kupffer cells), most reports on liver-mediated clearance of blood-borne virus and phage particles have uncritically claimed that the hepatic uptake of blood-borne virus/phages is by phagocytosis in Kupffer cells^9,12–14^. However, recent studies have shown that the liver sinusoidal endothelial cells (LSECs), lining the liver sinusoids and outnumbering the Kupffer cells in the sinusoids, are geared to clathrin-mediated endocytosis of small particles of the same size as most viral/phage particles, and thus play a major role in the elimination of blood-borne viral particles^10,15–18^. In fact, it has recently been shown that Adenovirus 1 (AdV), BK and JC polyomavirus-like particles, and HIV-like particles are rapidly cleared from the mouse circulation by uptake mainly in the LSECs^10,16,18^. However, the intracellular processing of virus and viral-like particles in these cells is currently unknown.

Knowing that phages easily gain access to the blood circulation, and that intravenously administered phages and other viruses are quickly and effectively cleared by the liver^9,10,16,17^, we hypothesize that bacteriophages that reach the liver are cleared via uptake and degradation by the LSECs. To test this hypothesis, we used GFP-labelled lysozyme-inactivated T4 bacteriophages (GFP-T4-phages)^19^ as a model virus to study viral endocytosis and metabolism in the LSECs. The T4 phage is a structurally simple, double-stranded DNA virus, and a perfect tool for various applications including cellular imaging^20,21^. Here, we studied the uptake and degradation of T4-phages in primary cultures of freshly isolated rat LSECs, demonstrating the transport of the particles to the lysosomes in real-time using deconvolution microscopy. The cellular binding and degradation of the phages were assessed by quantitative uptake studies using radiolabeled GFP-T4-phages. This is the first report showing *in vitro* uptake and lysosomal degradation of bacteriophages by LSECs, providing a direct evidence for the role of LSECs in bacteriophage clearance, entailing their contribution to the anti-viral defence mechanism.

## Results and Discussion

**Figure 1:**
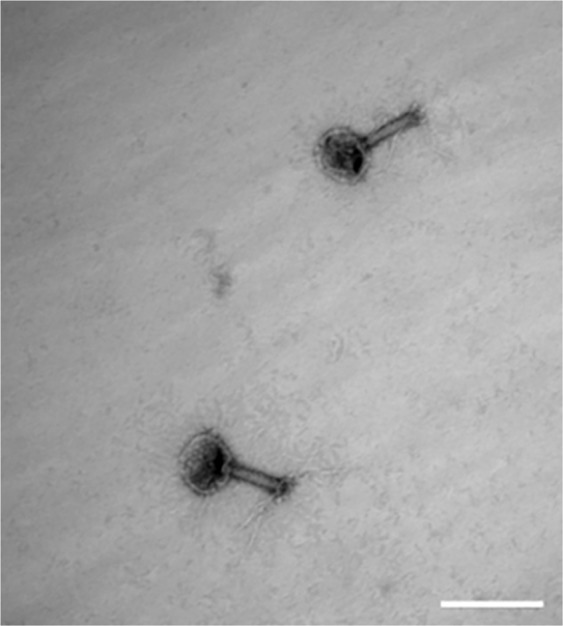
Transmission electron micrograph of T4-GFP. GFP-T4-phages diluted in PBS were placed on formvar-coated copper grids and negatively stained using uranyl acetate, prior to imaging using transmission electron microscopy. The phages were intact and found as single particles. Scale bar = 200 nm.

**Figure 2:**
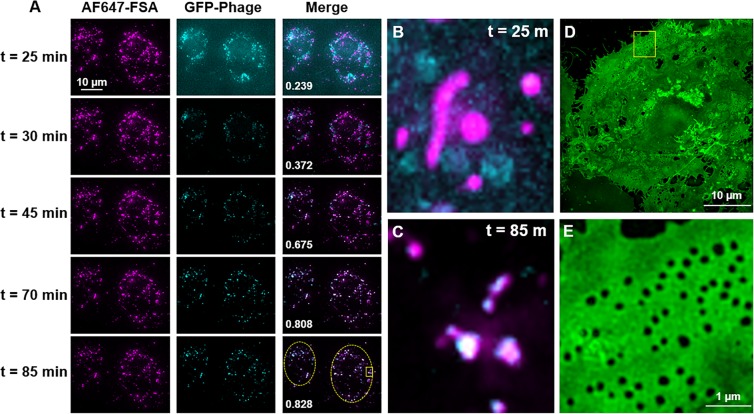
Time-lapse endocytosis of GFP-T4-phages by LSECs. (**A**) Rat LSECs preincubated with AF647-FSA were challenged with GFP-T4-phages, and endocytosis imaged in real-time by deconvolution microscopy (DV), every 5 min for about 90 min. Images represent maximum intensity projections of an 8 µm 3D z-stack. Colocalization values for FSA and phages for each time point (shown in merged images) are Pearson’s correlation coefficients calculated using the Costes threshold in Volocity Quantitation software. Only cell-containing regions of the image were used for colocalization analysis (dashed lines in t = 85 m, merged image). The results are representative of experiments performed with cells isolated from 2 animals, 3 culture dishes, and 10 fields of view including an approximate total of 150 cells. (**B**,**C**) DV maximum intensity projections of the highlighted inset at min 25 and min 85 of acquisition, respectively. (**D**,**E**) SIM image and its corresponding magnified inset of the plasma membrane of a representative live LSEC labelled with CellMask Green at the end of the acquisition time. Images represent maximum intensity projections of a 2 µm 3D SIM z-stack.

**Figure 3:**
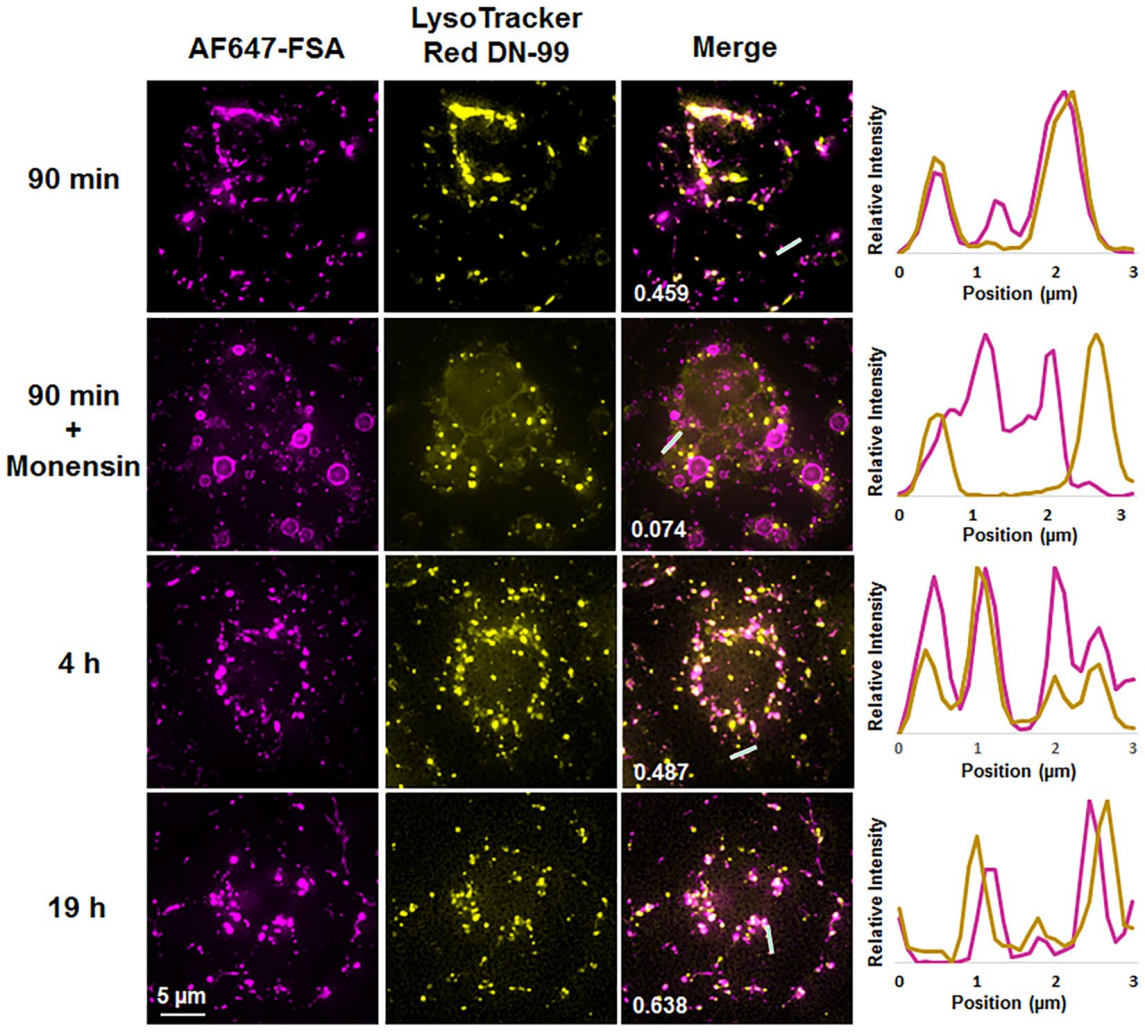
Time-lapse endocytosis of AF747-FSA in the presence or absence of monensin and LysoTracker. LSEC cultures were preincubated with LysoTracker (yellow) and challenged with AF647-FSA (magenta) in the absence or presence of monensin (10 µM). The uptake was monitored in real-time by DV overnight. Colocalization values for FSA and LysoTracker (shown in merged images) are Pearson’s correlation coefficients calculated using the Costes threshold in Volocity Quantitation software. Images represent maximum intensity projections of 4–9 µm 3D z-stacks. Right panel shows randomly selected line profiles within the cells, indicating the degree of colocalization of the two probes. The results are representative of experiments performed with cells isolated from 4 animals, and included 7 culture dishes, 49 fields of view, and approximately 350 cells; of which 2 animals, 2 culture dishes, 17 fields of view, and approximately 100 cells were in the presence of monensin).

Viruses are quickly (minutes) and extensively (>90%) eliminated by the liver, with LSECs in particular being the primary site of uptake, leaving only a small fraction of circulating virus to infect the body^9,10,16,17^. However, little is known about what these nanoparticles undergo once they enter the scavenging LSECs. Here we investigated the uptake of bacteriophages by primary cultures of rat LSECs, focusing on the clearance ability of the LSECs rather than viral infection. T4 bacteriophages were used as a model virus, which we genetically engineered to express the green fluorescent protein (GFP) in the capsid^19^ in order to allow live cell imaging of interaction of the phages with the cells. The integrity of the phages was confirmed by negative staining and transmission electron microscopy showing that the head of the phage was attached to the contractile tail (Fig. 1). Importantly, the phages were not aggregated, but found as single particles, a critical prerequisite for their recognition by LSECs, and not by the Kupffer cells which engulf larger complexes (>200 nm)^22,23^. Freshly isolated rat LSECs in culture were pulsed for 15 min with a low concentration of Alexa Fluor-647-formaldehyde treated bovine serum albumin (AF647-FSA) (5 µg/ml), non-attached ligand washed off, and the cells further incubated for another 1.5 h to functionally mark the late endosomal and lysosomal compartments^24^. The cells were then challenged with GFP-T4-phages and imaged in real time using deconvolution microscopy (DV), with 5-min intervals for 60 min from the time the phages were detected intracellularly (Fig. 2 and Supplementary Video 1). Due to low GFP-fluorescence intensity per phage particle, the DV was unable to resolve individual phage particles. As a consequence, the phages could not be detected during the initial phase of uptake (not shown). It was only after 20–25 min, when phages had clustered in the endosomal compartment, that the accumulated fluorescence signal was sufficient to resolve and thus visualize the phages. Few GFP-T4-phages were found colocalized in the same vesicles as AF647-FSA at 25 min post-incubation (Fig. 2A,B), after which the phages displayed a gradual accumulation in the same compartments as the AF647-FSA (Fig. 2A–C). These compartments are late endosomes/lysosomes (Fig. 3). These findings are in line with previously reported endocytosis of FITC-labelled ligands in rat and pig LSECs, where shortly after internalization and during the first 20 min, the ligands were found mostly in early endosomes, and some (approximately 23% in rat) in late endosomes, and by 2 h all were transferred to late endosomes^25–27^.

At the end of the incubation period, the plasma membrane of the live cell culture was labelled with CellMask Green (CMG), and a subset of the same cells that were studied using DV were then imaged at super resolution using structured illumination microscopy (SIM). This allowed us to confirm that the GFP-T4-phage containing cells were indeed LSECs due to the expression of fenestrations (Fig. 2D,E), the signature morphological characteristic of LSECs^28^. The fenestrations are small transmembrane pores with approximate diameters of 50 to 150 nm which provide open channels between the sinusoidal blood and the subendothelial space, facilitating the transfer of substrates between the blood and the parenchymal hepatocytes.

FSA is a frequently used and highly specific endocytic ligand for the LSEC scavenger receptors stabilin-1 and stabilin-2^29,30^, and we chose to use AF647-conjugated FSA here as a late endosome/lysosomal marker for live cell imaging. Previous studies using unconjugated and fluorescently conjugated FSA have shown that the ligand is avidly endocytosed by LSECs *in vivo* and *in vitro*, and transported to the lysosomes where it starts to be degraded within 10 min of cellular uptake^31,32^. When conjugated with fluorescent adducts (i.e. FITC or TRITC), or with gold particles, FSA follows the same route of uptake and degradation as unconjugated FSA, except that the non-degradable adducts remain trapped intralysosomally in LSECs for at least 24 h after uptake^25–27^. AF647 adduct acts in a similar way when bound to carrier ligands (i.e. albumin or dextran), as shown previously using other cell types^33,34^. Of note, in contrast to the present study, all previous studies assessing intracellular localization of endocytosed ligands in LSECs were performed by fixing the cells after various times of incubation with the ligands (pulse-chase studies). Currently, only two types of markers have been used in live cells to specifically label lysosomes for time lapse imaging. These are (i) LysoTracker probes, which are weak basic amines that accumulate in the acidic lumen of lysosomes^35^, and (ii) fluorescently labelled dextran and gold-labelled bovine serum albumin, both ligands that enter the cells via endocytosis and accumulate in the late endosomes/lysosomes^36,37^. In our setup, we found that LysoTracker® Red DND-99 (LT) used at the recommended concentration of 50–75 nM was susceptible to rapid photobleaching (data not shown). To bypass this limitation, we increased five-fold the LT concentration, allowing us to capture approximately 5–6 images of the lysosomal dye before photobleaching. However, high concentrations of LT and prolonged live cell imaging are known to affect the lysosomal pH that will sometimes radically disturb the intensity and spectral characteristics of a fluorophore^35,38^. In contrast, FSA, the formaldehyde-modified BSA, is a non-toxic and frequently used marker for LSECs, that is rapidly taken up by these cells at high specificity and efficiency^39^. Furthermore, FSA is easily tagged with fluorescent dyes. On this basis we incubated LSECs with a low concentration of AF647-FSA (5 µg/ml) for only 15 min, after which the non-bound ligand was washed off and the cells imaged. Given the very high endocytic efficiency of LSECs, visible uptake of this marker ligand is achieved at low ligand dose and short incubation period, avoiding non-specific uptake in all cells other than LSECs. In the present work this was a prerequisite for optimal imaging, avoiding overloading of the cells with fluorescence. AF647-FSA gradually accumulated in the lysosomes and remained colocalized with the LT up to 20 h of live imaging (Fig. 3). To further confirm that AF647-FSA can serve as a functional marker for different endocytic compartments, we used monensin as a tool to stop the internalization of the FSA at the early endosomes. Monensin is an ionophore that exchanges protons for Na^+^ and K^+^ thus perturbing gradients across cellular membranes, causing multiple effects on the intracellular transport of a variety of ligands, e.g. acidification of early endosomes, inhibition of receptor recycling back to the cell surface, inhibition of receptor-ligand dissociation and further transport of ligands to late endosomes/lysosomes^40^. In the presence of monensin, the AF647-FSA was arrested in fluorescent ring-like structures indicative of early endosomes^25^ (Fig. 3). No colocalization of the ligand with the lysosomes was further observed. This is consistent with previous reports of uptake of other ligands by LSECs in the presence of monensin^24,41,42^. With these experiments, we demonstrated that AF647-FSA used in live cell imaging could serve three purposes: i) as a specific functional marker for endocytically active LSECs, ii) as a marker for endosomal/lysosomal integrity, and iii) as a non-toxic and highly photostable marker for time-lapse imaging in live LSECs.

The final step of intracellular processing of ligands that have been taken up by LSECs is normally degradation in the endo-lysosomal compartment^39^. However, current fluorescence microscopy approaches lack the ability to probe the degradation of cargo within the cell. A previous study on peritoneal macrophages challenged with T2 phages, a close relative of T4 phages, attempted to detect degraded phages using transmission electron microscopy^43^. This “searching for the needle in the haystack” approach in the referred study did not succeed to show evidence of phage degradation. Other studies have used radioactive isotopes such as ^51^Cr, ^131^I, ^35^S or ^125^I to label T4 and M13 bacteriophages and polyoma JC and BK virus-like particles (VLPs)^9,10,44^. While these studies investigated the *in vivo* clearance mechanisms of these phages and viruses, showing that the liver is the major organ for uptake and degradation, they did not provide details on degradation at the cellular level. However, Simon-Santamaria *et al*.^10^ found degradation products in the blood circulation after intravenous injection of ^125^I-labeled JC polyoma VLPs, as well as uptake of non-labelled VLPs and intact virus in mouse LSECs *in vitro*, strongly suggesting that the LSECs were responsible for their degradation.

**Figure 4:**
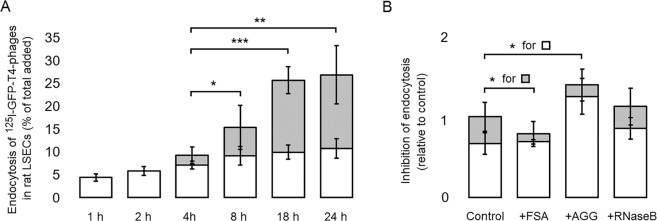
Endocytosis of T4-phages in LSECs *in vitro*. (**A**) Time course endocytosis of ^125^I-GFP-T4-phages by LSECs. LSEC cultures were incubated with radiolabelled phages for various time periods. For each time point, 3 separate wells containing cells, and 1 cell-free well were used. After each time period, the supernatant from the cells and cell-free well was collected along with one 0.5 ml washing volume of PBS. Trichloroacetic acid (TCA) precipitation was then used to differentiate between free iodine = degraded phages (TCA soluble) (grey columns), and unbound, intact phages (TCA precipitable). Cell bound and internalized phages were quantified in the cell lysates, after solubilizing the cells in 1% SDS (white columns). The results were normalized by subtracting the amount of radioactivity corresponding to the non-specific binding and free ^125^I in cell-free wells. Each experiment was performed in triplicates, on cells isolated from four animals (Total N = 12 cell cultures for each time point). Bars represent mean ± SD. *p < 0.05, **p < 0.01, ***p < 0.001 represent the statistical differences between the total endocytosis at 18 h and 24 h as compared to 4 h. (**B**) The specificity of uptake was studied by incubating the cells with ^125^I-GFP-T4-phages in the absence (Control) or presence of blocking concentrations (0.1 mg/ml) of FSA, ribonuclease B (RNaseB) and aggregated gamma globulin (AGG), inhibitors for stabilin1/2, mannose receptor and FcγRIIb2, respectively. Cell association and degradation were assessed as above. The results are presented as relative uptake compared to the control which was set to 1. Each experiment was performed in triplicates, on cells isolated from 3 animals (Total N = 9 cell cultures for each time point). Bars represent mean ± SD.

To investigate the intracellular processing of the bacteriophages in LSECs in the present study, we challenged the cells *in vitro* with trace amounts of ^125^I-GFP-T4-phages. This enabled us to measure the rate of uptake and degradation over a time interval of 24 h. The functional integrity of LSECs was confirmed in these experiments by the ability of the cells to endocytose and degrade ^125^I-FSA (not shown). We found that the LSECs rapidly internalized the ^125^I-labeled GFP-T4 phages, supporting our imaging data presented in Fig. 2, and that increasing amounts of degradation products were released to the spent culture medium shortly after the phages had reached the late endosomes/lysosomes (Fig. 4A). Bacteriophages are relatively large viral particles, with a complex structure composed of hundreds of proteins, and highly stable under a variety of harsh environmental conditions. Their catabolism undoubtedly takes longer time than single proteins e.g. modified albumin and collagen alpha chains, known to be efficiently eliminated by LSECs^24,32^ (Fig. S4), and is more dependent on the high concentration and specific activity of enzymes in the lysosomes, the terminal degradative compartment^24,45^. Nevertheless, during 24 h post incubation of LSECs with T4-phages, 27% and 16% of total added phages were internalized and degraded by the cells, respectively, demonstrating a substantial capacity of LSECs to take up and degrade the phages.

Using a variety of receptors (mannose receptor, scavenger receptors, and the endocytic Fc-gamma receptor IIb2), the LSECs can directly recognize and internalize pathogen associated molecular patterns, cellular debris and immune complexes, contributing thus to the liver’s immune tolerance^46^. To gain further insights into the uptake mechanism of the T4-phages, we investigated whether the stabilin1/2, mannose receptor, or the FcγRIIb2 are involved in the uptake of T4-phages by incubating the cells with radiolabeled phages in the absence or presence of blocking concentrations of native, unlabeled FSA, RNaseB, and AGG, inhibitors for stabilin1/2, mannose receptor, and FcγRIIb2, respectively^31,47,48^. The results presented in Fig. 4B show a borderline significant (*p* = 0.0497) decrease of phage degradation in the presence of FSA as compared to control. Decreased degradation was also noted in the presence of AGG, although not significant (*p* = 0.17). Since these ligands had no inhibitory effects on the binding (rather the opposite in the presence of AGG), the results suggest that stabilin1/2 and FcγRIIb2 are probably not involved in the internalization of T4 phages in LSECs. The observed decrease in phage degradation is most probably due to FSA and AGG overloading the later stages of the endocytic pathway. No effect on the uptake and degradation was observed in the presence of RNase B either, suggesting that the mannose receptor is also not involved in the uptake T4 phages. Further studies are needed to investigate the mechanisms by which these cells recognize and internalize bacteriophages.

In conclusion, we have demonstrated that freshly isolated and functionally intact LSECs efficiently take up and degrade T4 bacteriophages at high capacity, indicating that these cells act as a primary anti-viral defence system, adding to the role that these cells have in innate immunity. *In vivo* studies have reported that injected bacteriophages used in phage therapy are quickly removed from the circulation, thus resulting in lower efficiency of the therapy^49–51^. Administration of huge doses of phages is frequently required to saturate unwanted liver uptake, showing the great virus elimination capacity of the liver^9,17^. Our study showing that the LSECs take up and degrade phages may explain some of the challenges with using bacteriophages both in phage therapy and as gene delivery vehicles.

## Materials and Methods

### Animals and ethics statement

Sprague Dawley, Crl:CD(SD), male rats (Charles River, Sulzfeld, Germany) were group housed (2–3 rats per cage) in conventional Eurostandard type IV cages with aspen bedding (Tapvei, Estonia) and with nesting material (Sizzelnest®, Datesand, UK), rat tunnels (Scanbur, Norway) and aspen chew blocks (Scanbur, Norway) as environmental enrichment. The rats were housed under controlled environmental conditions (21°C ± 1°, relative humidity 55% ± 5% and 12 h light/12 h dark cycle). They were fed a standard chow *ad libitum* (RM1-E, Special Diet Service, UK) and tap water ad libitum. All methods were carried out in accordance with relevant guidelines and regulation protocols, and approved by the Norwegian Food Safety Authority (approval ID: 8455). All experimental protocols and animal handling were approved by and carried out according to local authorities (Department of Comparative Medicine). The rats (body weight 150–300 g) were anesthetized with a mixture (ZRF-mix) of zolazepam /tiletamine hydrochloride 12.9/12.9 mg/mL (Zoletil forte vet, Virbac, Norway), xylazine 1.8 mg/mL (Rompun, Bayer Nordic, Norway) and fentanyl 10.3 µg/mL (Actavis, Norway).

### Chemicals and materials

Collagenase was from Worthington Biochemical Corporation (Lakewood, NJ, USA). Iodogen was from Pierce Chemicals (Rockford, IL, USA), and carrier-free Na^125^I from PerkinElmer Norge AS (Oslo, Norway). Bovine serum albumin (BSA) was from MP Biomedicals (Solon, OH, USA). Fibronectin was purified from human plasma by affinity chromatography on Gelatin Sepharose 4B as described by the manufacturer (GE Healthcare, Uppsala, Sweden). Percoll and PD-10 columns (Sephadex G-25) were from GE Healthcare. Serum-free Roswell Park Memorial Institute (RPMI-1640) cell culture medium (supplemented with 20 mM sodium bicarbonate, 0.006% penicillin, and 0.01% streptomycin) and monensin were from Sigma-Aldrich (Oslo, Norway). Culture dishes of 35 mm diameter and #1.5 glass coverslip-bottom were from MatTek Corp. (Ashland, MA, USA). LysoTracker Red DND-99, CellMask Green, and AlexaFluor-647 carboxylic acid were from ThermoFisher Scientific (Oslo, Norway). Trichloroacetic acid (TCA) was from Merck (Darmstadt, Germany). Formaldehyde-treated bovine serum albumin (FSA) was prepared by treating BSA with 10% formaldehyde in 0.2 M carbonate buffer, pH 10, for 3 days as described^52^.

### Preparation and GFP labelling of T4 phages

GFP-Enterobacteria phages T4 (GFP-T4-phages) were prepared as previously described^19^, dialyzed against phosphate buffer saline (PBS) and stored aseptically at 4 °C at a titre of 10^8^ PFU/ml.

### Isolation of liver sinusoidal endothelial cells (LSECs), and assessment of endocytosis and specificity of uptake

LSECs were isolated and purified from anesthetized rats as described^53^, and seeded at a density of 0.5 × 10^6^ cells/cm^2^ on fibronectin coated coverslip-bottom dishes (MatTek *In Vitro* Life Science Laboratories, Bratislava, Slovak Republic), or 24 well tissue culture plates (Sarstedt, Nümbrecht, Germany) in serum-free RPMI-1640. All experiments started 2–3 h after isolation and seeding. For quantitative studies of uptake and degradation, confluent cultures of freshly isolated LSECs established in 24-well culture dishes coated with fibronectin were incubated in 0.2 mL RPMI containing 3–4 × 10^4^ cpm ^125^I-GFP-T4-phages. Time-course endocytosis was performed by incubating the cells for 1, 2, 4, 8, 18, and 24 h. For each time point, 3 separate wells containing cells, and 1 cell-free well were used. At the end of each time point, the percent of degraded phages was measured by collecting the spent medium together with one wash volume of 0.5 mL PBS. TCA (0.75 mL, 20%) was added to precipitate intact phages. The amount of TCA-soluble radioactivity measured in the supernatant after centrifugation represented degraded phages. To determine the amount of cell bound and internalized phages, the cells were lysed in 2 × 0.5 mL of 0.1% sodium dodecyl sulfate (SDS) in PBS. The radioactivity was measured in all three fractions using a Cobra II, Auto-Gamma detector (Packard Instruments, Laborel, Oslo, Norway). The results were normalized by subtracting the amount of radioactivity corresponding to the non-specific binding and free ^125^I in cell-free wells. The specificity of uptake was studied by incubating the cells with ^125^I-GFP-T4-phages in the absence or presence of blocking concentrations (0.1 mg/ml) of FSA, ribonuclease B (RNaseB) or aggregated gamma globulin (AGG), inhibitors for stabilin1/2, mannose receptor and FcγRIIb2, respectively.

### Fluorescence labelling and radioiodination

GFP-T4-phages in PBS were labelled with carrier-free Na^125^I, using Iodogen as described by the manufacturer (Pierce Chemicals), and separated from unbound ^125^I on a PD-10 column. The resulting specific radioactivity was approximately 40 × 10^6^ cpm per 10^6^ PFU phages. FSA was conjugated with Alexa Fluor-647 carboxylic acid succinimidyl ester according to the manufacturer’s instructions (ThermoFisher Scientific). Free AF647 was separated from FSA using a Vivaspin 6, 10.000 kDa cutoff (ThermoFisher Scientific). The final concentration of the protein was measured with a NANODROP 2000 spectrophotometer (ThermoFisher Scientific).

### Imaging methods and analyses

#### Transmission electron microscopy (TEM)

Glow discharged formvar-coated copper grids were placed on top of 5 µl droplets of GFP-T4-phages (stock solution of 10^8^ PFU/ml in PBS was diluted 1/10 in 0.9% sodium chloride), for 5 min in a moist chamber. The grids were gently washed on droplets of double distilled water and incubated for 20 seconds on a droplet of freshly made 1% uranyl acetate in double distilled water. The uranyl acetate solution was partly removed by filter paper, and the grids air dried, with the remaining uranyl acetate solution faced up, before electron microscopy. The images were recorded in a JEOL JEM 1010 transmission electron microscope (TEM) (JEOL ltd, Tokyo, Japan), operating at 80 kV, and equipped with Morada digital CCD camera (Olympus, Tokyo, Japan). The analysis of the negatively stained GFP-T4-phages showed that the phages were intact and found as single particles (Fig. 1).

#### Deconvolution and structured illumination microscopy

After washing to remove non-adherent cells, cultures on coverslips were pre-incubated for 30 min with 0.25 μM LysoTracker in RPMI, then with 5 μg/mL AF647-FSA for 15 min, in the presence or absence of 10 μM monensin. The non-bound FSA was removed by washes with PBS, and the cells allowed to endocytose the FSA for another hour in culture medium containing LysoTracker with or without monensin. For live cell imaging, the LSECs were incubated with 0.3 mL RPMI containing 5 × 10^6^ PFU GFP-T4-phages, and the uptake followed by deconvolution (DV) microscopy with 5 min intervals between acquisitions, over a period of 90 min. The experiments were terminated by live staining of the plasma membrane with CellMask Green (1:1000 in RPMI) and imaging using structured illumination microscopy (SIM). Live cell imaging was performed using a DeltaVision OMX V4 Blaze imaging system (GE Healthcare) equipped with a 60 × 1.42 NA oil-immersion objective (Olympus), 3 sCMOS cameras, a solid-state illumination source for widefield deconvolution imaging, and a 488 nm laser for structured illumination imaging. Additional widefield deconvolution imaging was performed on a DeltaVision Elite microscope (GE Healthcare) equipped with an identical 60 × 1.42 NA oil-immersion objective (Olympus), single sCMOS camera, and a solid-state illumination source. Widefield deconvolution images were acquired in 4–8 μm z-stacks with one image taken per 0.20–0.25 μm z-step. SIM image stacks of 2 μm were acquired with 15 raw images (five phases, three angles) acquired at each plane with a *z*-step size of 0.125 μm. During live imaging, the cells were kept under an incubator/control chamber supplied by GE Healthcare, calibrated to approximately 5% humidified CO_2_, and a temperature set at approximately 36 °C. This paper incorporates the results of imaging experiments from approximately 20 different cell isolations. Over the course of the study, experimental procedures were refined and/or had to be repeated/optimized due to high fluorescence background, improvement of staining, quality of the primary cell cultures, etc. Typically, 5–10 fields of view were imaged for each experiment with 2 (for SIM) or 10 (for DV) cells per field of view. 1–6 imaging experiments were conducted for each cell isolation, including controls and photobleaching assessments (Supplementary Fig. S3).

The experimental conditions are listed in Supplementary Table. Raw datasets were computationally reconstructed and colour alignment was performed using SoftWoRx software (GE Healthcare) for both deconvolution and structured illumination images. Colocalization was analysed in Volocity 6.3 (PerkinElmer) using the Costes threshold to compute the Pearson’s correlation coefficient, for hand-selected image regions which only contained a cell (no cell-free background regions). For clarity of display, figure images were linearly adjusted for brightness and contrast using Fiji (https://fiji.sc, version 2.0.2.)^54^.

#### Statistical analysis

For all endocytosis experiments, the statistical analyses were performed using the Excel 2016 software (Microsoft, USA). Student’s *t* test (two tailed, unpaired) was used for statistical analysis. All experiments were performed in triplicate, on cells isolated from 3 animals (Total N = 9 cell cultures for each time point and/or inhibitor) and the results were presented as mean ± standard deviation. The significance was set at: non significant *p* > 0.05, significant *p < 0.05, **p < 0.01, and ***p < 0.001.

## Supplementary information

Supplementary information.

SI Video.
