# Supplementary material for: Liver sinusoidal endothelial cells contribute to the uptake and degradation of entero bacterial viruses
Source: Sci Rep. 2020 Jan 21;10:898. doi: 10.1038/s41598-020-57652-0 (PMC6972739; doi:10.1038/s41598-020-57652-0)

Supplementary information

Liver sinusoidal endothelial cells contribute to the uptake and degradation of entero bacterial viruses

Cristina I. Øie[1#¶*], Deanna L. Wolfson[1¶], Tanji Yasunori[2], Gianina Dumitriu[3], Karen K. Sørensen[3], Peter A. McCourt[3], Balpreet S. Ahluwalia[1&], Bård Smedsrød[3&]

S1 Video. Time lapse GFP-Phage (green) accumulation in the same endocytic compartments as FSA (red) (uploaded online)

S2 Table. Experimental conditions under deconvolution and structured illumination microscopy. Excitation and emission ranges take into account both the polychroic mirror and filters.

Figure	Instrument	Modality	Fluorophore	Excitation			Emission	Z-stack (μm)	Timelapse
				λ (nm)	Transmission	Exposure (ms)	λ (nm)		
2A,B,C	OMX	Deconvolution	AF647-FSA	639-652	10 %	7	664-702	8	13 x 5 min
			GFP-Phage	462-492	50 %	100	505-549		
2D,E	OMX	Structured Illumination	CellMask Green	488	10 %	15	505-549	2	
3 (90 min)	DV Elite	Deconvolution	AF647-FSA	625-643	10 %	30	662-696	4	
			LysoTracker-99	531-556	5 %	50	575-611		
3 (Monensin)	OMX	Deconvolution	AF647-FSA	639-652	50 %	35	664-702	8	
			LysoTracker-99	562-580	31,30 %	10	592-627		
3 (4 h)	DV Elite	Deconvolution	AF647-FSA	625-643	10 %	50	662-696	6,6	
			LysoTracker-99	531-556	32 %	40	575-611		
3 (19 h)	DV Elite	Deconvolution	AF647-FSA	625-643	10 %	70	662-696	7,2	
			LysoTracker-99	531-556	32 %	200	575-611		

S3 Figure. Assessment of photobleaching - The max and mean fluorescence intensity over time, normalized to value of 1. The acquisition settings are listed in the right panel.

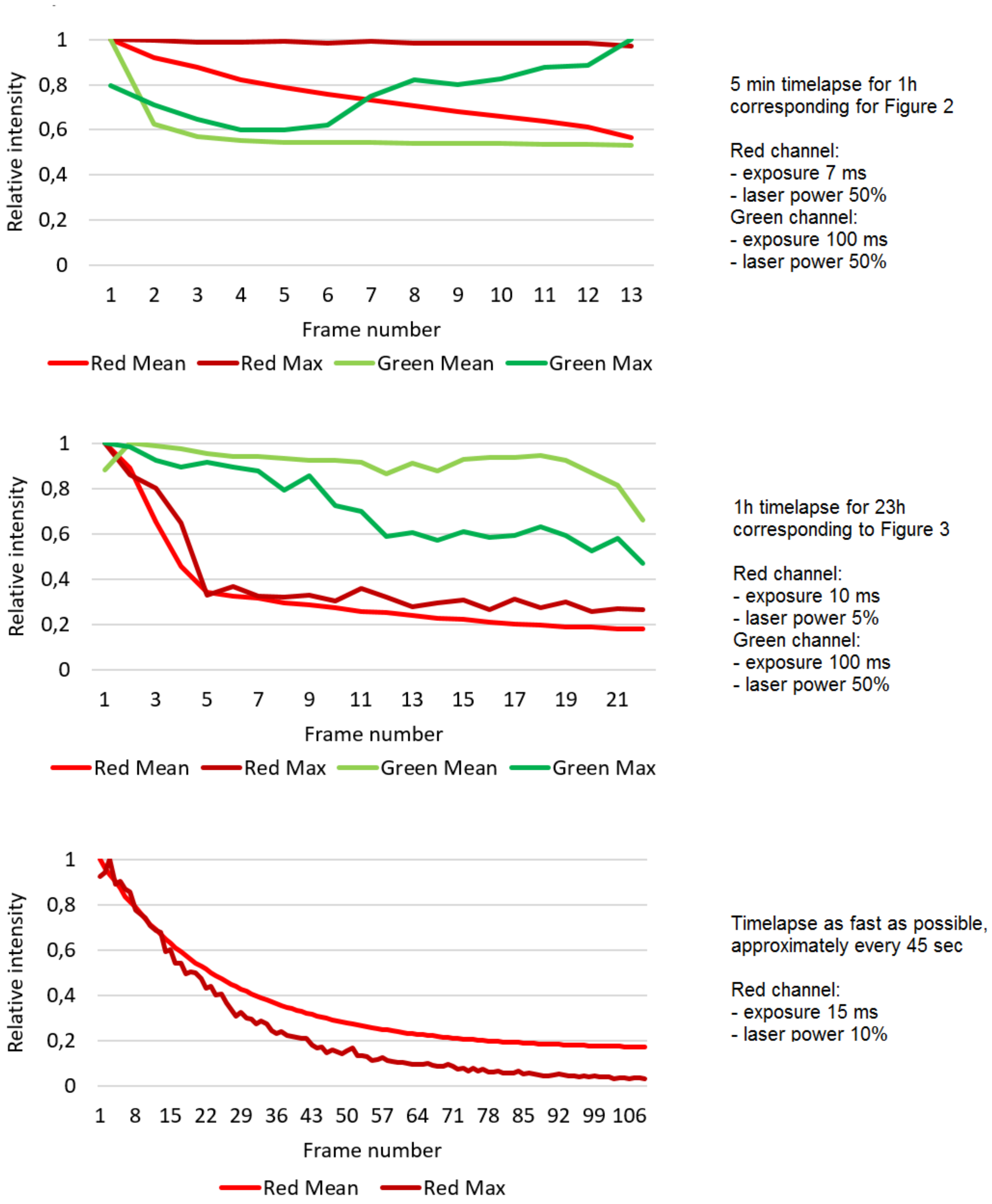

S4 Figure. **Uptake and degradation comparison between T4-phages and FSA in LSECs**

A) Time course endocytosis of ^{125}I-GFP-T4-phages and B) Time course endocytosis of ^{125}I-FSA by LSECs. LSEC cultures were incubated with ^{125}I-GFP-T4-phages or ^{125}I-FSA for the indicated time periods. For each time point, 3 separate wells containing cells, and 1 cell-free well were used. After each time period, the supernatant from the cells and cell-free well was collected along with one 0.5 ml washing volume of PBS. Tricholoacetic acid (TCA) precipitation was then used to differentiate between free iodine = degraded phages (TCA soluble) (grey columns), and unbound, intact phages (TCA precipitable). Cell bound and internalized phages and FSA were quantified in the cell lysates, after solubilizing the cells in 1% SDS (white columns). The results were normalized by subtracting the amount of radioactivity corresponding to the non-specific binding and free ^{125}I in cell-free wells. Each experiment was performed in triplicates, on cells isolated from four animals in A) (Total N=12 cell cultures for each time point), and 3 animals in B) (Total N=9 cell cultures for each time point). Bars represent mean ± SD. *$p < 0.05$, **$p < 0.01$, ***$p < 0.001$ represent the statistical differences between the total endocytosis at 18h and 24h as compared to 4h.

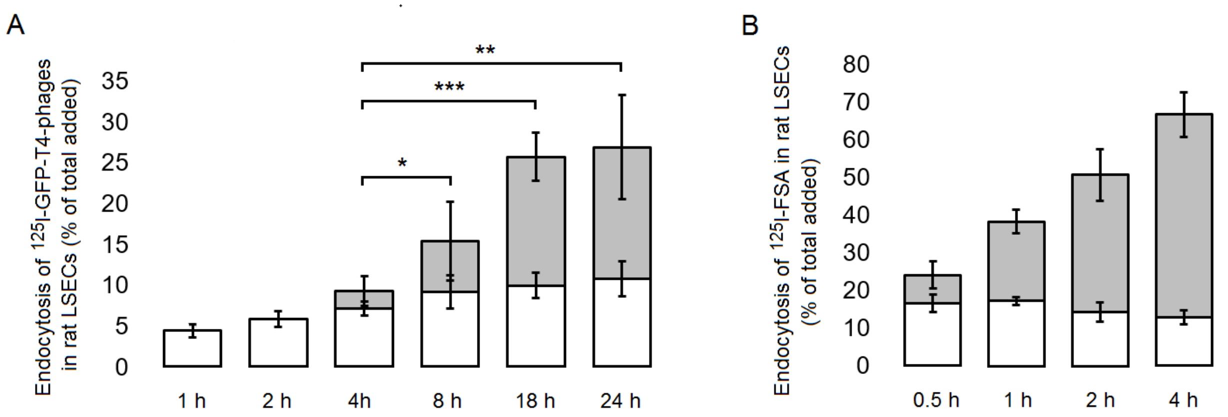

Supplement: Supplementary file 1 — Supplementary information. [file 41598_2020_57652_MOESM1_ESM.pdf]
